# Corn silk polysaccharides attenuate diabetic nephropathy through restoration of the gut microbial ecosystem and metabolic homeostasis

**DOI:** 10.3389/fendo.2023.1232132

**Published:** 2023-12-04

**Authors:** Wenting Dong, Yuanyuan Zhao, Xiuwei Li, Jinhai Huo, Weiming Wang

**Affiliations:** ^1^ School of Pharmacy, Harbin University of Commerce, Harbin, China; ^2^ Institue of Chinese Materia, Heilongjiang Academy of Traditional Chinese Medicine, Harbin, Heilongjiang, China

**Keywords:** diabetic kidney disease, corn silk polysaccharides, intestinal-kidney axis, metabonomics, gut microbiota

## Abstract

**Introduction:**

The pathogenesis of diabetic nephropathy (DN) is complex, inflammation is the central link among the inducing factors in the existing research, and the gutkidney axis could scientifically explain the reasons for the accumulation of chronic low-grade inflammation. As both a medicine and food, corn silk contains abundant polysaccharides. Historical studies and modern research have both confirmed its intervention effect on diabetes and DN, but the mechanism of action is unclear.

**Methods:**

In this study, a DN rat model was generated, and the therapeutic effect of corn silk polysaccharides (CSPs) was evaluated based on behavioral, histopathological and biochemical indicators. We attempted to fully understand the interactions between CSPs, the gut microbiota and the host at the systemic level from a gut microbiota metabolomics perspective to fundamentally elucidate the mechanisms of action that can be used to intervene in DN.

**Results:**

Research has found that the metabolic pathways with a strong correlation with CSPs were initially identified as glycerophosphate, fatty acid, bile acid, tyrosine, tryptophan and phenylalanine metabolism and involved Firmicutes, Bacteroides, Lachnospiraceae-NK4A136- group and Dubosiella, suggesting that the effect of CSPs on improving DN is related to changes in metabolite profiles and gut microbiota characteristics.

**Discussion:**

CSPs could be harnessed to treat the abnormal metabolism of endogenous substances such as bile acids and uremic toxins caused by changes in gut microbiota, thus alleviating kidney damage caused by inflammation. In view of its natural abundance, corn silk is safe and nontoxic and can be used for the prevention and treatment of diabetes and DN.

## 1 Introduction

Diabetic nephropathy (DN) is the most common and serious microvascular complication in diabetic patients, and it easily develops into end-stage renal disease compared to other causes. The clinical treatment of DN mostly adopts early intervention, which mainly focuses on controlling blood sugar and blood pressure first and then protecting renal function. Although this can delay the progress of DN, it cannot completely stop its progress. The pathogenesis of DN is complex and still being explored. Based on the current research results, DN is closely related to abnormal glucose metabolism, hemodynamics, the renin angiotensin aldosterone system, advanced glycation end products, inflammation and oxidative stress. It is worth noting that the key link between many factors lies in inflammation or oxidative stress, so it is particularly important to determine the causes of chronic low-grade inflammation (1, 2).

The gut microbiota is a complex ecosystem that has been described as the “second genome” controlling human health. It interacts with diseases through genes, intermediates, and metabolic activities. Its structural diversity and abundance directly impact human health, and current research suggests associations with a variety of diseases, including cardiovascular and cerebrovascular diseases, the immune system, digestive function, metabolism, and the nervous system. Studies in recent years have also confirmed that the imbalance of gut microbiota involves many links from diabetes to diabetic nephropathy, such as insulin secretion disorder, advanced glycation end products, chronic inflammatory state, etc. (3), and the identification of intestinal microflora may be used as a biomarker for diagnosing and predicting diseases (4, 5). However, the number of cells, bacteria and genes contained in the gut microbiota is very large, so its functional contribution to DN has not been systematically characterized. To clarify the relationship between the symbiotic system and diseases, it is necessary to identify not only the microbial composition but also the metabolites under the expected conditions to obtain a more comprehensive analysis, which will be more convincing and accurate. In other words, metabolites are the main compounds that connect the gut microbiota and the host, and they are small molecular substances that reflect the functional state of cells. It is very important and necessary to integrate the gut microbiota and metabonomics to fully understand the interaction between the gut microbiota and the host at the system level(6, 7). At present, it has been found that aromatic amino acids, choline, bile acids, uremic toxins, lipopolysaccharides and short-chain fatty acids are all key endogenous substances involved in the interaction between gut microbiota and diabetic nephropathy. Abnormal metabolism of these substances or obstruction of renal excretion can cause oxidative stress and inflammatory reactions.

Polysaccharides, as a type of macromolecule, play a key role in regulating host health, largely depending on the process of digestion, absorption and fermentation in the body (8). In fact, the physiological effects and digestion and absorption characteristics of polysaccharides determine their correlation with the gut microbiota. Polysaccharides, serving as the primary carbon source for gut microbiota, contribute to maintaining the balance of gut microbiota, stimulating the immune system, and preserving the integrity and health of the gut. Currently, the interplay between polysaccharides, gut microbiota, and metabolic disorders is becoming increasingly evident. Polysaccharides can significantly reduce chronic low-grade inflammation by balancing gut microbiota, leading to improvements in metabolic disorders such as blood glucose, obesity, and lipid metabolism. Corn silk is composed of the style and stigma of Z. mays L., which was introduced to China during the Ming Dynasty. It is believed to be a diuretic that can promote diuresis and reduce swelling, soothe the liver and promote bile flow, eliminate dampness and alleviate jaundice. The “Dian nan Materia Medica” and “Ling nan Caiyao Lu” have recorded the use of corn silk for the treatment of diabetes in detail, and “Modern Practical Chinese Medicine”, “Dictionary of Traditional Chinese Medicine”, “National Compilation of Chinese Herbs”, “Chinese Materia Medica”, “Clinical Chinese Medicine” and many other works on Chinese medicine have recorded the inclusion of corn silk as a medicine, which can be used for acute and chronic nephritis, edema, proteinuria, and diabetes. The research team utilized metabonomics methods to study the hypoglycemic mechanism of corn silk in the early stage. Among various potential biomarkers, corn silk significantly affected the metabolism of aromatic amino acids, choline, and bile acids. In addition, uremic toxins such as PCS and indole acetic acid were found in the metabolites with abnormal changes (9). This suggests that corn silk may modulate key metabolites associated with end-stage renal function failure, and these metabolites are closely linked to the gut microbiota. While both historical and contemporary research confirm the impact of corn silk on diabetes, the underlying mechanisms and active components remain unclear. Research on the intervention of diabetic nephropathy using corn silk polysaccharides (CSPs) is particularly scarce. Therefore, building upon previous research, we selected the active component CSPs, established a DN rat model, and introduced the concept of the gut-kidney axis. By elucidating the interaction among ‘gut microbiota-metabolites-kidney,’ we aimed to identify dominant bacterial strains strongly correlated with potential biomarkers. This approach may unveil a natural intervention method for the prevention and progression of diabetic nephropathy and enhance the recognition of the medicinal value of corn silk.

## 2 Materials and methods

### 2.1 Chemicals and reagents

Acetonitrile and methanol were obtained from Merck (HPLC grade, Darmstadt, Germany). Distilled water was purchased from Watson’s Food & Beverage Co., Ltd. (Guangzhou, Guangdong, China). Formic acid was acquired from Thermo Fisher Company (HPLC grade, Waltham, MA, USA). Streptozotocin (STZ) (lot number: S0130) was purchased from Sigma-Aldrich (St. Louis, MO, USA). Metformin hydrochloride tablets (Lot: ABW1966) were purchased from Sino-US Shanghai Squibb Pharmaceutical Co. Ltd.

### 2.2 Animals and feed

A total of 85 male SD rats weighing 200 ± 20 g were purchased from the Department of Laboratory Animal Science, Harbin Medical University (SCXK, 2019-001) (Harbin, Heilongjiang, China). The animals were fed for 1 week before the experiment to eliminate the stress response and adapted on a 12 h light-dark cycle (22 ± 2°C with a relatively constant humidity of 50 ± 10%) under barrier system facilities. Ordinary rodent chow and a high sugar and fat feed were purchased from BEIJING KEAO XIELI FEED CO., LTD (Lot:2021060402, Beijing, China). The study was conducted strictly according to the ethical guidelines for using experimental animals in Heilongjiang Province and was guided and approved by the animal ethics committee of the Academy of Traditional Chinese Medicine of Heilongjiang Province ([2011]93).

### 2.3 CSPs preparation

Corn silk was collected from the experimental field (Latitude: 47.348079° N; Longitude: 123.95348° W) of the Qiqihar branch of Heilongjiang Academy of Agricultural Sciences in September 2022, and identification of corn silk (Stigma maydis L.) was performed by Wang Wei-Ming (Heilongjiang Academy of Chinese Medicine Sciences). Extraction of corn silk decoction: 10 kg of corn silk was added to 15 times the amount of water and boiled three times for 2 hours each time. After boiling, the three extracts were combined, filtered and concentrated to a density of 1.25. Slowly add 95% ethanol to the concentrated corn silk extract, stirring continuously, until the ethanol content reaches 80%. The mixture was stored at 4°C overnight and the precipitate at the bottom was collected. The precipitates were extracted by adding 10 times the amount of petroleum ether or acetone, and the Sevage method was used to remove proteins. Before freeze-drying, a 3000 kDa molecular weight cutoff dialysis bag was used three times.

A UV - VIS spectrometer (TU-1901, Persee General Instrument Co., Beijing, China) was used to scan CSPs in the range of 200-800 nm. The CSPs was scanned by a Fourier transform infrared spectrometer (Perkin Elmer, Shelton, CT, USA) in the range of 4000 to 400 cm^−1^. The molecular weight of CSPs was identified by high-performance gel permeation chromatography (HPGPC), which was performed on a Shimadzu LC-20AT series system (Kyoto, Japan) coupled with an evaporative light scattering detector (ELSD, Agilent Technologies Co. Ltd., USA). Chromatography was performed on a TSK gel G4000PWXL column (7.8 × 300 mm, Tosoh Corporation, Japan). The molecular weight was calculated by a calibration curve of standard dextran with molecular weights ranging from 4 kDa to 2,000 kDa. Then, CSPs were derived using PMP (0.5 M) and NaOH (0.3 M) at 70°C for 100 min. The CSPs were analyzed by HPLC on an Agilent 1100 Infinity system equipped with an Agilent Extend-C18 column (250 mm × 4.6 mm, 5 μm) with detection by a UV detector. The eluent used was acetonitrile-0.02 M ammonium acetate solution (15: 85, v/v) with a flow rate of 1 mL/min and a temperature of 25°C. By the above detection, the properties of CSPs were preliminarily determined.

### 2.4 Establishment of the DN rat model and drug administration

Referring to the modeling approach in the literature (10, 11), 85 rats were randomly divided into two groups, the normal group (NC) and the model group, with the NC fed a basal diet (n = 12) and the model group fed a high sugar and high fat diet (n = 73). After 4 weeks, all rats were fasted for 12 hours without water. The model group was injected with 35 mg/kg STZ solution via intraperitoneal injection, while the NC was injected with the same volume of citric acid buffer solution. After 72 hours, blood was taken from the tail tip to measure FBG. Rats with FBG values greater than 16.7 mmol/L that remained stable for two weeks were selected and randomly divided into five groups according to FBG, with 12 rats in each group: diabetic nephropathy (DN), metformin (MET), the low dose of polysaccharides (PL), the medium dose of polysaccharides (PM) and the high dose of polysaccharides (PH). Rats in each group were injected intraperitoneally with 35 mg/kg STZ solution weekly except for NC. NC and DN were given distilled water by gavage at 20 mL/kg, metformin was administered via gavage at a dosage of 0.25 g/kg, and the dosage concentrations of polysaccharides, from low to high, were 100 mg/kg, 200 mg/kg and 400 mg/kg by gavage for 8 weeks of continuous intervention. During the course of the study, behavioral observations were conducted.

### 2.5 Kidney weight, kidney index

The kidneys of rats in each group were weighed, and the kidney organ index was calculated according to the kidney weight/body weight. The left kidney was fixed in 4% paraformaldehyde for pathological observation, and the right kidney was quenched in liquid nitrogen and transferred to a refrigerator at -80°C for later study.

### 2.6 FBG, lipid level and renal function

After 8 weeks of administration, all rats were fasted for 10 hours, and FBG levels were measured from the tail tip. Then, the rats were placed into metabolic cages and fasted for 12 hours, during which urine samples were collected to detect ACR. After anesthesia, blood was taken from the abdominal aorta, and the blood samples were centrifuged at 4°C and 3000 r/min ^-1^ for 10 min. The supernatant was collected, and Scr, TC, TG, HDLC, and LDLC were detected using an automatic biochemical analyzer (7600, Hitachi, Japan).

### 2.7 Inflammatory cytokines and oxidative stress markers detection

An ELISA assay kit was used to detect the levels of inflammatory mediators IL-6 and TNF-α. The levels of oxidative factors SOD and MDA in serum were measured using extraction and TBA (thiobarbituric acid) methods, respectively.

### 2.8 Histopathological observation of the kidney

The fixed kidney tissue was dehydrated, embedded, sectioned, HE and Masson stained, and sealed, and the tissue sections were observed in detail under a microscope. The collagen pixel area and the corresponding tissue pixel area were measured in each section using Image-Pro Plus 6.0 analysis software uniformly using the pixel area pixel as the standard unit, and the collagen area percentage= collagen pixel area/tissue pixel area was calculated.

### 2.9 16S

The DNA was extracted from the samples using the CTAB method, and the DNA concentration and purity were checked on a 1% agarose gel. The DNA was then diluted to a concentration of 1 ng/µL using sterile water. The 16SV4 region was amplified using barcode-specific primers (515F-806R), the mixed PCR products were purified using the QIAquick gel extraction kit, and the sequencing library was generated using the TruSeq^®^ DNA PCR-Free library builder kit. The library was evaluated on a Qubit@ 2.0 fluorometer and an Agilent 2100 Bioanalyzer and finally sequenced on the NovaSeq 6000 platform. After splicing and filtering the original data, operational taxonomic unit (OTU) clustering and species classification analysis were carried out based on the effective data. The significant differences between groups of the microbial community were discussed by using the t-test method. After obtaining the 16S sequencing results, Tax4Fun and PICRUSt were used for functional prediction of the community.

### 2.10 Pretreatment of samples for metabolomics studies

Two hundred microliters of serum was added to 800 μL of precooled methanol, vortexed for 2 min, left for 10 min, and centrifuged for 10 min at 4°C and 13000 r/min. Eight hundred microliters of the supernatant was dried with nitrogen, redissolved in 200 μL of 80% methanol, and centrifuged to obtain the supernatant. One milliliter of urine from each group of rats was filtered through a 0.22 μm filter membrane, and the filtered solution was collected for injection. Two processed serum and urine samples were taken from each group and mixed together as quality control (QC) samples. Five consecutive QC samples were collected before establishing Sequence, and after every 12 sample injections, 1 QC sample was injected for testing. To determine the stability of the detection system, in addition to examining the clustering of QC samples, the eight peaks with high response intensity were also selected from the QC samples, and the RSD values of retention time and peak area were calculated.

### 2.11 UPLC-Q-TOF/MS

Liquid phase conditions: Waters Acquity UPLC HSS BEH C18 Chromatography column (1.7 μm, 2.1 mm × 100 mm) with AQUITY UPLC HSS BEH C18 VanGuard Pre-Column (1.7 μm, 2.1 mm × 5 mm) for the analysis of serum samples. A Waters Acquity UPLC HSS T3 C18 Chromatography column (1.7 μm, 2.1 mm × 100 mm) with an AQUITY UPLC HSS T3 C18 VanGuard Pre-Column (1.7 μm, 2.1 mm × 5 mm) was used for the analysis of urine samples. The column temperature was maintained at 35°C, and all the samples were maintained at 4°C during the analysis. The mobile phases were composed of water (A) and acetonitrile (B), each containing 0.1% formic acid. The gradient elution program of serum was set as follows: 0~5 min, 5% ~ 70%B; 5~10 min, 70% ~ 80%B; 10~12 min, 80% ~ 100%B; 12~14 min, 100%B; 14~14.1 min, 100% ~ 5%B; 14.1~18 min, 5%B. The gradient elution program of urine was set as follows: 0~3 min, 5% ~ 10%B; 3~9 min, 10% ~ 30%B; 9~14 min, 30% ~ 100%B; 14~15 min, 100%B; 15~15.1 min, 100% ~ 5%B; 15.1~18 min, 5%B. A flow rate of 0.3 mL/min was used. The flow rate was 0.3 mL/min, and the injection volume was 5 μL.

Using an electrospray ionization source, the scans were conducted in both positive and negative ion modes. The ion source voltage was set at 5500V for positive ions and -4500V for negative ions. Additionally, the ion source temperature was maintained at 550°C. Other parameters include: declustering potential, 80 V/–80 V; collision energy, 35 eV/–35 eV; collision energy spread, 15 eV/–15 eV; the nebulizing gas is N2, auxiliary gas 1, 55 psi, auxiliary gas 2, 55 psi, and the curtain gas is 35 psi; mass range, 80-1200 m/z. IDA was set to perform secondary mass spectrometry scanning on the top 8 peaks with response values exceeding 100 cps, production scan, 50–1,500 m/z. The raw data were acquired using Analyst TF 1.6 software (5600, AB SCIEX, USA).

### 2.12 Statistical analysis

The research results of the biochemical indicators were analyzed using the intergroup t-test in SPSS 20.0 software. P<0.05 was considered to indicate a statistically significant difference. All the LC-MS raw files were converted using Progenesis QI 2.3 software (Waters, Milford, USA) for data processing. Automatic aligning, peak picking and normalization of the chromatogram peak in the sample were performed. EZINFO 3.0.3.0 software was used to perform principal component analysis (PCA) and orthogonal projection to latent structures discrimination analysis (OPLS-DA). From the OPLS-DA, the potential biomarkers were selected based on the variable importance in projection (VIP) values (VIP>1) with the t test results (P<0.05). By matching to the Human Metabolome Database (HMDB: http://www.hmdb.ca/) and METLIN (http://metlin.scripps.edu), structural identification was carried out.

After the initial determination of the molecular formula and molecular weight of compounds, the molecular formulas of all compounds were imported into Peakview 2. 0 (AB ScieX, USA), focusing on ppm values, isotopic abundance ratios, and secondary mass spectra. The comparison was performed to increase the accuracy of the endogenous metabolites that were searched for.

## 3 Results

### 3.1 Characterization of CSPs

**Figure 1:**
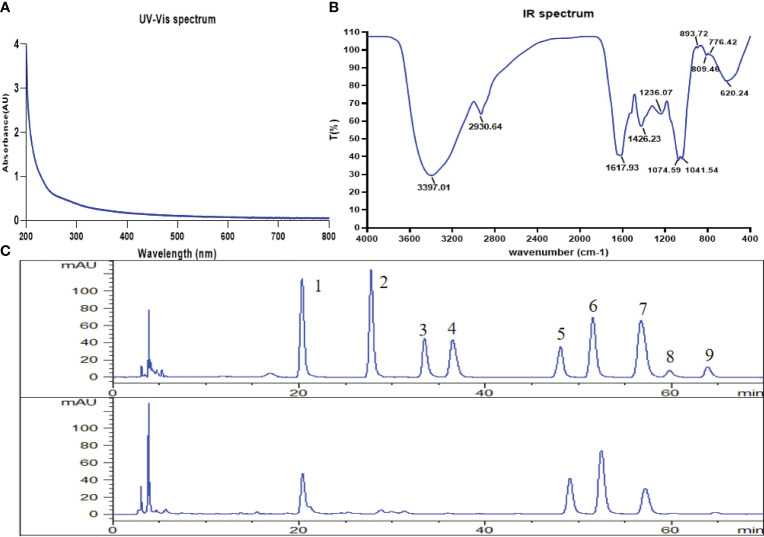
Structural characterization of CSPs. **(A)** UV spectrum, **(B)** FT-IR spectrum, **(C)** the HPLC chromatograms of standard monosaccharides and CSPs (from top to bottom). 1. D-mannose, 2. L-rhamnose, 3. D-glucuronic acid, 4. D-galacturonic acid, 5. D-glucose, 6. D-galactose, 7. L-arabinose, 8. D-xylose, 9. L-fucose.

The UV spectrum showed no absorption at 260 nm and 280 nm, indicating that the CSPs had no protein or nucleic acid (**Figure 1A**). The FT-IR spectrum of CSPs showed a strong broad band at approximately 3397 cm^−1^ (**Figure 1B**), which was attributed to the stretching vibration of O-H bonds (12). The C-H stretching and bending vibrations led to weak bands at 2930 cm^−1 [12]^. The two peaks at 1617 cm^−1^ and 1426 cm^−1^ were attributed to the bound water (13). The strong peaks at approximately 1041 cm^−1^ were the characteristic absorption of C-O-C and C-O-H of the pyranose ring (13). Furthermore, the peak at 894 cm^−1^ was a β-type glycosidic bond, and the peak at 776 cm^−1^ was due to the symmetrical stretching vibration of pyranose (14). CSPs showed three peaks on HPGPC-ELSD. According to the standard curve, t_R_ = -0.2836 log M_w_ + 25.71 (M_w_: weight-average molar mass; t_R_: retention time), with a high correlation of R^2 = ^0.9946, the average molecular weights of the CSPs were calculated as 2.72 × 10^7^ Da, 2.13 × 10^5^ Da and 4.91 × 10^4^ Da. Monosaccharide composition analysis displayed that consisted of D-mannose, glucose, galactose and arabinose with a molar ratio of 25.86: 55.18: 52.43: 23.31 (**Figure 1C**).

### 3.2 General observation of animals

The rats in the NC had a good mental state and normal fur, and body weight continued to increase. The rats in the DN group became less active, and their fur gradually turned yellow and lost its shine. They also started eating more food, drinking more water, and producing more urine (as observed from the increased frequency of changing their bedding and the comparison of urine volume collected from metabolic cages) and smelled like rotten apples. The above mentioned status of the rats in the CSPs administration group was superior to that of the model group.

### 3.3 Kidney weight and kidney index

**Figure 2:**
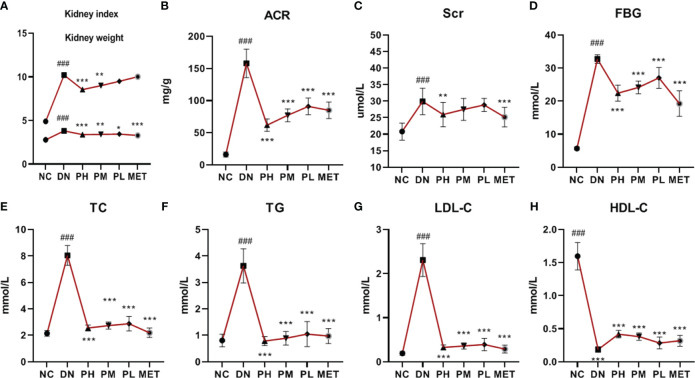
Changes in kidney function **(A–C)**, FBG and lipid metabolism **(E–H)** of rats in each group. ^###^ *P*<0.001 vs. NC; ***P<0.001 vs. DN, **P<0.01, *P<0.05 vs. DN. **A** (Kidney weight/index), **B** (ACR), **C** (Scr), **D** (FBG), **E** (TC), **F** (TG), **G** (LDL-C), **H** (HDL-C).

Compared with NC, the kidney weight and kidney index of DN were significantly higher. After treatment with CSPs, the kidney weight and kidney index were significantly reduced in a dose-dependent manner. PH showed the most significant effect (P<0.001). The effect of MET on reducing the kidney weight was more significant than that of PH, possibly due to the lower body weight in MET, resulting in less noticeable changes in the kidney index (P > 0.05) (**Figure 2A**).

### 3.4 The effect of CSPs on kidney function in DN rats

Compared with the NC, the ACR of the rats was significantly increased in the DN (P < 0.001), suggesting a significant increase in urinary protein levels in the DN. Compared with the DN, the ACR values were significantly lower in each CSPs dose group (P<0.001). The upward trend of blood creatinine of the rats in the DN group suggested that kidney function had changed (P<0.001), and only the PH group showed a decreasing trend (P<0.01) in ACR levels, but the intensity was lower than that in the MET group (**Figures 2B, C**).

### 3.5 FBG and blood lipid levels

This section examines the effects of CSPs on FBG and blood lipids in rats. FBG, TC, TG and LDL-C levels were found to be significantly higher (P<0.001) and HDLC levels were significantly lower (P<0.001) in the DN, indicating that the rats with diabetic nephropathy developed a disorder in glucose and lipid metabolism. After treatment with different doses of CSPs, a significant and dose-dependent regulatory effect was observed (P<0.001). Compared with MET, PH showed slightly less improvement in FBG (**Figure 2D**), TC (**Figure 2E**), but demonstrated a stronger effect in reducing TG (**Figure 2F**). Meanwhile, based on the results, it can be observed that PH shows a less significant improvement in LDL-C (**Figure 2G**) compared to HDL-C (**Figure 2H**) in the blood.

### 3.6 The anti-inflammatory and anti-oxidant effects of CSPs

**Figure 3:**
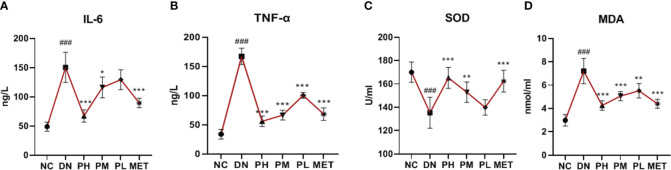
Effects of CSPs on inflammatory factors **(A, B)** and oxidative stress **(C, D)** indexes in DN rats. ^###^ *P*<0.001 vs. NC; ***P<0.001, **P<0.01, *P<0.05 vs. DN. **A** (IL-6), **B** (TNF-α), **C** (SOD), **D** (MDA).

Compared with the NC, the IL-6 and TNF-α levels of the rats was significantly increased in the DN (P<0.001), indicating a significant inflammatory response *in vivo*. The expression levels of IL-6 and TNF-α were significantly reduced after CSPs intervention, with TNF-α showing a more pronounced antagonistic effect in a dose-dependent manner. Metformin also exhibited a significant anti-inflammatory effect, the effect in the high-dose group was similar to that of metformin, as shown in **Figures 3A, B**. In addition to the inflammatory response, rats in the DN also demonstrated oxidative stress, as evidenced by decreased in SOD levels (P<0.001) and increased in MDA levels (P<0.001). All doses of CSPs exhibited significant anti-oxidant effects (**Figures 3C, D**).

### 3.7 Kidney histopathological sections

**Figure 4:**
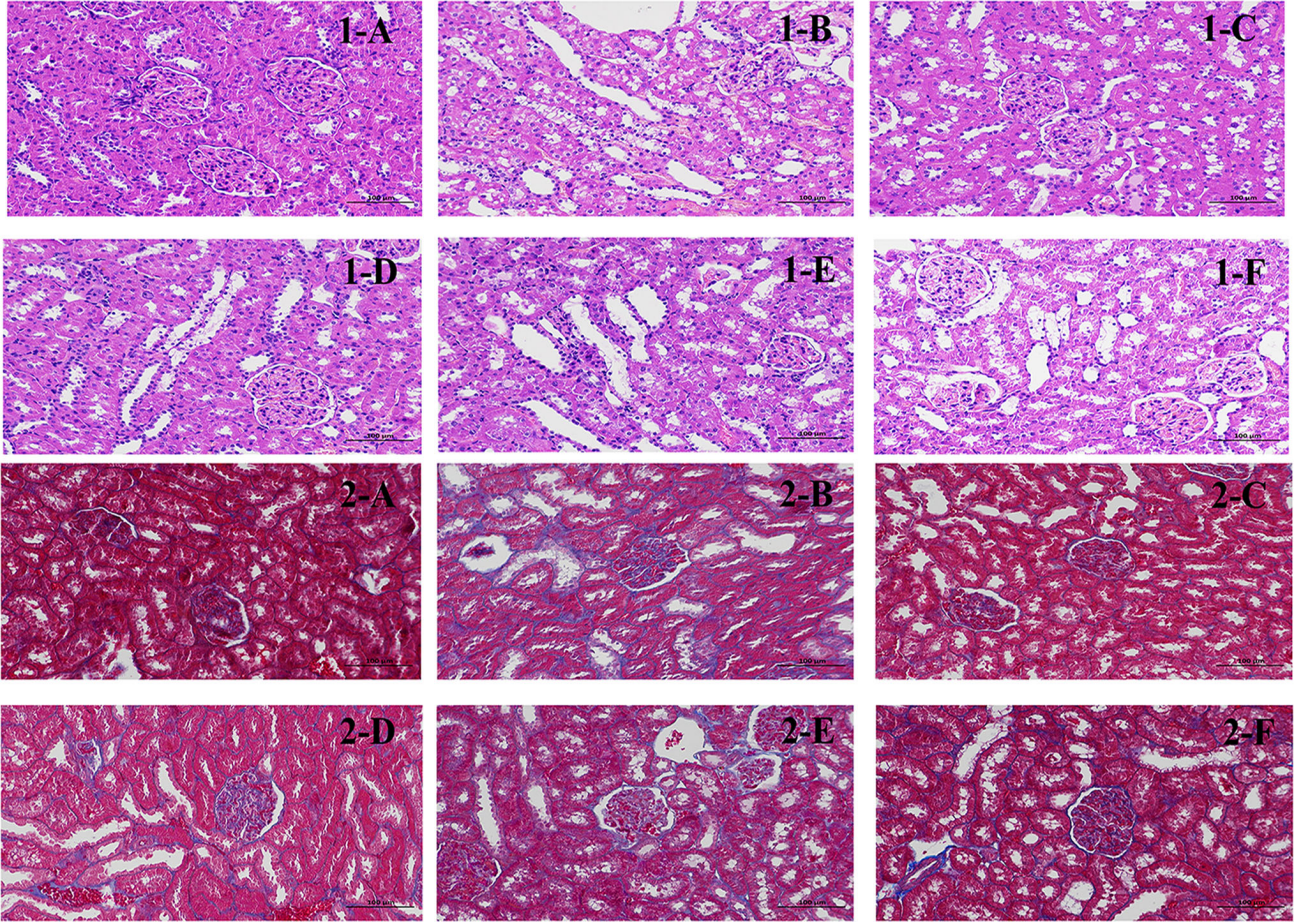
Effects of CSPs on pathological sections of kidney tissue in DN rats. 1: HE staining, 200×; 2: Masson staining, 200×. (A) NC, (B) MC, (C) PH, (D) PM; (E) PL; (F) MET.

The staining results showed (**Figure 4**-1) that the glomeruli in the cortex of the NC were evenly distributed, the number of cells in the glomeruli and the stroma were uniform, the tubular epithelial cells in the renal cortex were rounded and full, the brush border was neatly and regularly arranged, and no obvious inflammatory changes were observed. In the DN, a large number of tubular epithelial cells with aqueous degeneration, swelling, cytoplasmic laxity and vacuolization, nuclei solidification and deep staining, and tubular dilatation could be seen. The presence of lymphocytic infiltration is visible in renal tissue (arrows). After treatment with CSPs, the matrix of the kidneys appeared more uniform, and the number of dilated tubules significantly decreased. Additionally, there was a reduction in swelling and degeneration of epithelial cells, and no lymphocytic infiltration was observed. The degree of improvement was dependent on the dose of CSPs. Compared to DN, there was also a significant improvement with metformin, but in terms of pathological changes, the degree of improvement was not as high as that of PH.

Masson staining results showed that collagen fibers were stained blue, which was used to show the staining method of inflammatory factors and fibers. Glomerular and tubular basement membrane and collagen fibers were stained blue, which may serve as a straightforward way to evaluate kidney tissue damage (**Figure 4**-2). The results are expressed as the percentage of collagen area (%), with the collagen area percentage in the NC being approximately 0.45329, and a significant increase in the collagen area percentage was observed in the DN, which was 8.08322. Compared to the DN, CSPs can significantly reduce the collagen content, with PH 1.86331, PM 2.83223, PL 3.86671. Metformin had a slightly better collagen area percentage than PL and approached that of PM.

### 3.8 Alpha and Beta diversity

A total of 66 samples (n=11) were collected in this experiment, and after quality filtering and chimera removal by QIIME 1.9.1 software, 75,439 valid tags were obtained for final use in the subsequent analysis, and sequences with ≥97% similarity were clustered into the same OTUs using Uparse software. A total of 2,239 OTUs were obtained, and the OTU abundance information was normalized for alpha diversity and beta diversity analysis. The results showed that the coverage indices of each group were greater than 99%, indicating that most of the bases in the genome of the tested sample could be sequenced. The observed species values showed that the number of species in the DN was significantly reduced. PH and PM had a significant recovery effect. However, the level of recall observed in PL and MET was relatively low. This result showed the same trend of variation in the Chao1 and ACE indices, suggesting that CSPs could increase the abundance of abnormally varying bacterial populations. Finally, it can be clearly seen from the Shannon index that the species distribution uniformity of the gut microbiota in the DN was significantly reduced, and there was a significant recovery effect in the PH and the PM. The above results are shown in **Table 1**.

**Table 1:** Alpha diversity index ( $\bar{\text{x}}\pm \text{s}$ , n=11).

	observed_species	chao1	ace	shannon	goods_coverage
NC	1115.09±32.139	1177.07±34.683	1179.48±32.414	7.51±0.146	0.99820± 0.000131
DN	1044.00±25.144###	1105.75±29.583###	1113.35±27.008###	7.07±0.401##	0.99814±0.000192
PH	1148.55±43.332***	1211.41±56.667***	1214.63±54.434***	7.57±0.205**	0.99815±0.000271
PM	1119.91±45.971***	1179.14±52.453***	1182.41±53.586**	7.48±0.248**	0.99822±0.000194
PL	1034.36±45.280	1094.34±49.227	1098.30±48.700	7.10±0.316	0.99825±0.000289
MET	1052.40±52.709	1119.12±53.549	1118.79±52.176	7.14±0.187	0.99817±0.000103

^###^ P <0.001, ^##^P <0.01vs NC;^***^P <0.001,^**^ P <0.01 vs DN.

**Figure 5:**
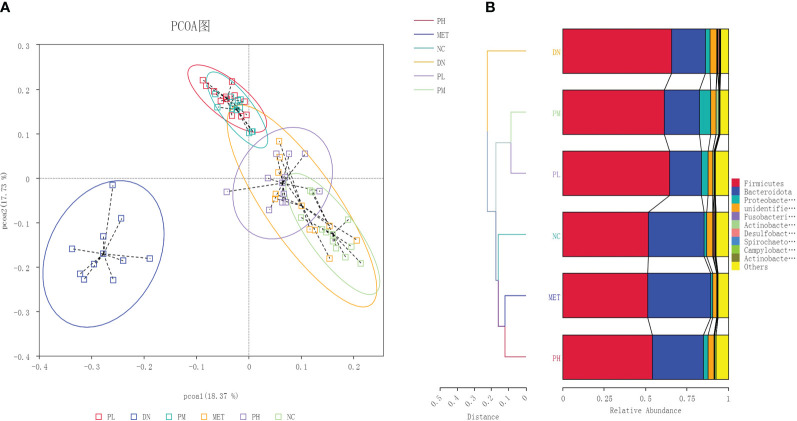
The PCoA **(A)** and NMDS **(B)** analysis plots of the gut microbiota in each group of rats.

β-diversity was used to examine the similarity of species composition between different groups of communities. The results were shown in **Figure 5**. DN was obviously separated from NC, indicating that the microbial diversity was significantly changed. After the administration of treatment, the clustering position of CSPs on the PCoA plot was significantly away from the DN and closer to the NC, indicating that CSPs can significantly recover the abnormal changes in microbial diversity. UPGMA clustering can clearly show the closer two groups, and it can be seen from the **Figure 5** that PH and MET first clustered with NC, then clustered with PM and PL, and finally clustered with DN, indicating that CSPs has a recall effect.

### 3.9 Differential species at different taxonomic levels

**Figure 6:**
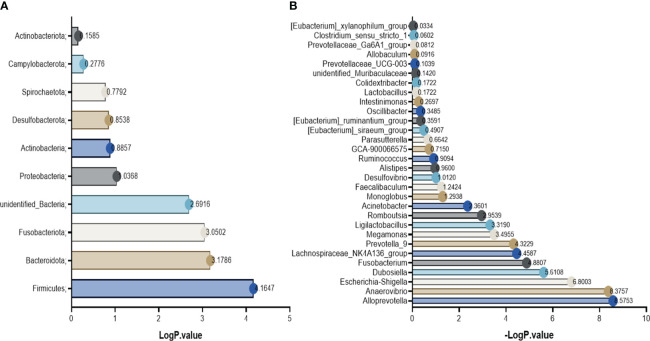
Differential bacteria between groups. **(A)** the phylum level; **(B)** the genus level.

Based on the annotation results and abundance information of species in each group, species in the top 10 at the phylum level and top 30 at the genus level were selected using the maximum value sorting method, and species with significant differences (P<0.01, -logP>2.0) were identified by the t-test between groups (**Figure 6**). From the results, it can be seen that the phylum with abnormal changes in rats with DN were Firmicutes, Bacteroidota and Fusobacteriota. The change of genus were Ligilactobacillus, Lachnospiraceae_NK4A136_group, Dubosiella, Romboutsia, Prevotella-9, Alloprevotella, Megamonas, Escherichia-Shigella, Anaerovibrio, Acinetobacter, Fusobacterium.

### 3.10 Serum and urine metabonomics

**Figure 7:**
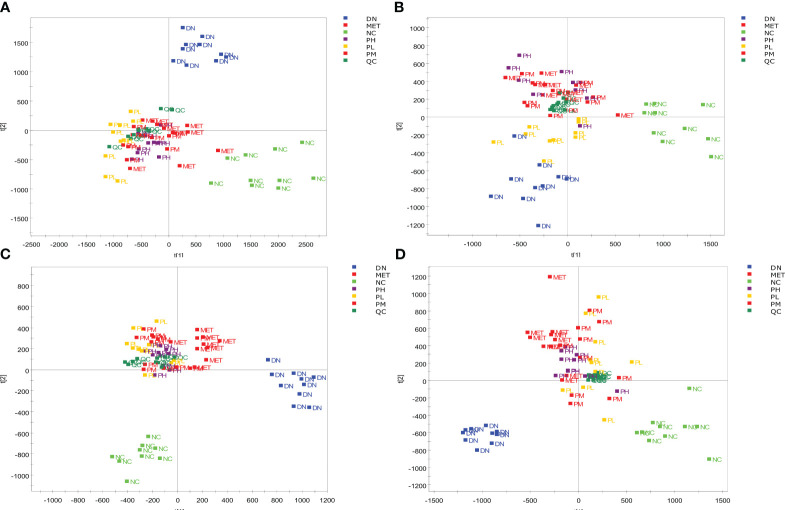
PCA score of the serum and urine samples from each group of rats. Serum **(A)** in positive ion mode; Serum **(B)** in negative ion mode; Urine **(C)** in positive ion mode; Urine **(D)** in negative ion mode.

After data processing and analysis, it was found that the QC samples were well clustered in the positive and negative ion modes (**Figure 7**), and 8 different m/z were randomly selected for Rt and Area RSD value calculation. It was found that the RT RSD value ≤ 2.99 and the peak area RSD value ≤ 8.85, which indicated that the system was stable with reliable values, and the results are shown in **Table 2**. The PCA plots of serum and urine of rats in each group showed (**Figure 7**) that the samples were well clustered within the NC, DN group and CSPs group, and there was a significant dispersion between the groups, which clearly showed that the metabolic profile was far from the DN and tended to be closer to the NC after CSPs treatment, indicating that CSPs could positively regulate the metabolic disorder in DN rats.

**Table 2:** RSD values of the RT and peak area of QC samples in positive and negative ion mode.

ESI+	Serum-RSD			urine-RSD		
	m/z	Rt	Area	m/z	Rt	Area
	637.3048	0.08	6.76	338.0869	1.21	7.64
	524.3704	0.1	8.24	431.0979	0.42	7.49
	508.375	0.09	7.61	447.0922	0.19	6.79
	570.355	0.07	7.51	200.2368	0.04	7.75
	429.2825	0.14	6.15	461.108	0.38	6.57
	355.2632	0.16	7.65	566.4277	0.23	3.87
	604.3528	0.35	5.74	220.1173	2.93	7.68
	388.2541	0.58	7.35	367.1511	2.74	5.71
ESI-	303.2334	0.13	7.75	297.0985	0.43	7.65
	327.2335	0.12	8.16	338.0886	0.72	8.38
	480.3086	0.13	8.64	336.0726	1.06	8.05
	554.3462	0.09	5.84	315.0721	2.99	7.82
	319.2295	0.05	5.84	242.0134	1.02	5.06
	407.2809	0.08	8.1	269.0452	0.05	8.6
	464.3031	0.13	8.3	162.0566	0.56	8.7
	297.0989	0.26	7.53	308.1173	1.98	8.85

**Figure 8:**
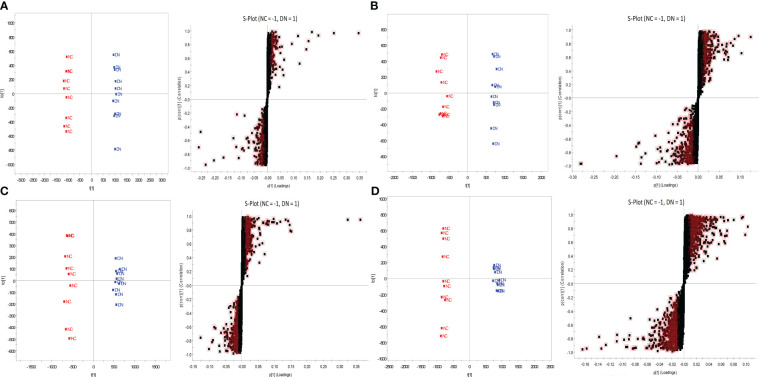
OPLS-DA score plot and S-plot of NC and DN. Serum **(A)** in positive ion mode; Serum **(B)** in negative ion mode; Urine **(C)** in positive ion mode; Urine **(D)** in negative ion mode.

OPLS-DA was performed on the metabolic profiles of serum samples and urine samples from the NC and DN groups, and a score plot with S-plot loadings was obtained (**Figure 8**), which showed that the NC was clearly separated from the DN, indicating that the metabolic profiles of serum samples and urine from DN rats were significantly changed. In the S-plot, metabolites with VIP>1 and P<0.05 were screened as potential biomarkers, which were identified using HMDB, METLIN and other databases. Using PeakView software, 47 and 57 potential differential metabolites were finally identified in the urine and blood of DN rats based on molecular weight, molecular formula, and MS/MS spectra, and the results are shown in **Supplementary Tables 1**, **2**.

### 3.11 Metabolic pathway analysis

**Figure 9:**
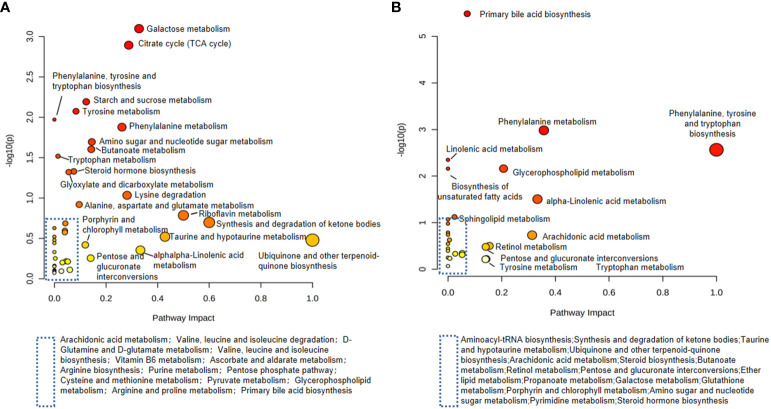
Metabolic pathway analysis in renal tissue and urine. **(A)** Urine metabolic pathways associated with DN; **(B)** Serum metabolic pathways associated with DN.

The potential metabolites found in urine and serum were imported into the MetaboAnalyst 5.0 website separately for pathway analysis. The results showed that a total of 40 metabolic pathways were enriched in urine samples and 29 metabolic pathways in serum samples. Several metabolic pathways were found to be same, like Phenylalanine metabolism, Steroid hormone biosynthesis, Galactose metabolism, Tyrosine metabolism, Phenylalanine, tyrosine and tryptophan biosynthesis, Amino sugar and nucleotide sugar metabolism, Tryptophan metabolism, Arachidonic acid metabolism, alpha-Linolenic acid metabolism, Primary bile acid biosynthesis, Biosynthesis of unsaturated fatty acids, Glycerophospholipid metabolism, etc. (**Figure 9**).

**Figure 10:**
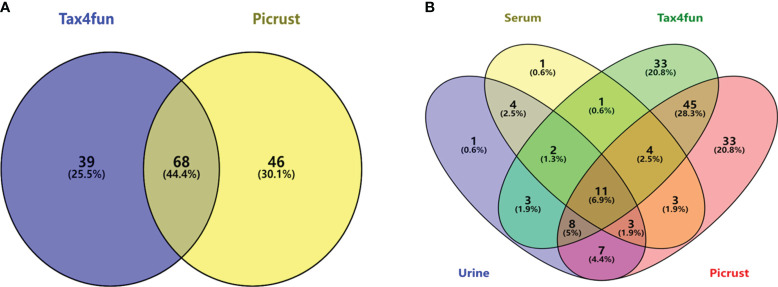
KEGG pathway prediction for gut microbiota based on two databases **(A)**; the intersection of enriched metabolic pathways with metabolomics **(B)**.

Currently, the main functional prediction databases for gut microbiome 16S amplicons are PICRUSt and Tax4Fun, both of which are similar but have different emphases. Tax4Fun focuses on predicting the functional potential based on the OTUs that are already annotated in KEGG pathways, while PICRUSt places more emphasis on ancestral prediction. To more accurately determine the KEGG pathways enriched by gut microbiota, the intersection of the two prediction results was taken based on the NC and the DN group. The results showed that 110 KEGG pathways could be predicted for Tax4Fun, while 117 KEGG pathways could be predicted for PICRUSt. The intersection of the two groups resulted in 68 KEGG pathways, as shown in **Figure 10**.

Venny analysis was performed to identify the intersecting pathways between the predicted KEGG metabolic pathways of the gut microbiota and the metabolomics results (**Figure 10**). A total of 11 common pathways were identified, including alpha-linolenic acid metabolism, arachidonic acid metabolism, biosynthesis of unsaturated fatty acids, butanoate metabolism, galactose metabolism, glycerophospholipid metabolism, phenylalanine, tyrosine and tryptophan biosynthesis, primary bile acid biosynthesis, synthesis and degradation of ketone bodies, tryptophan metabolism, and tyrosine metabolism.

### 3.12 Identification of dominant microbial species and metabolites

**Figure 11:**
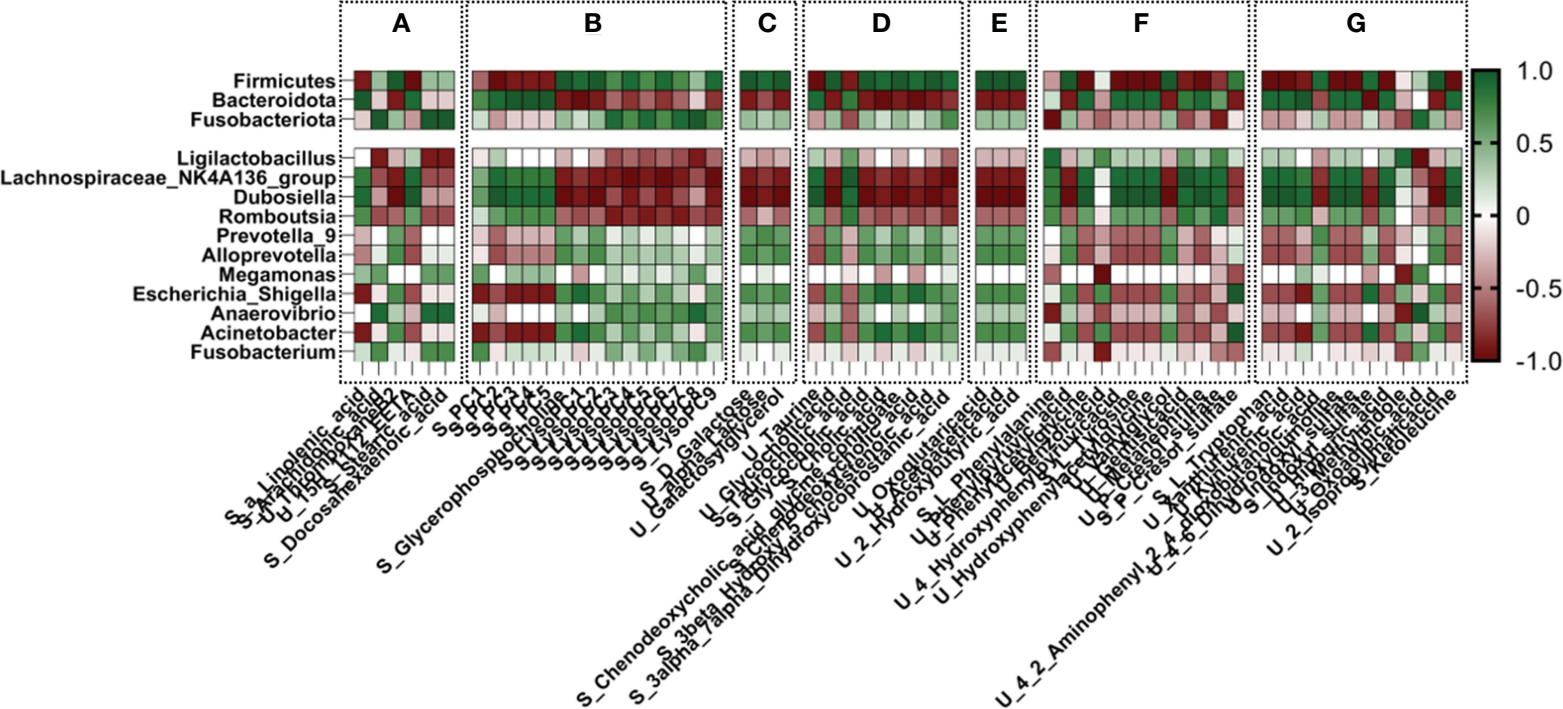
Correlation analysis of gut microbiota with differential metabolites in urine and serum. **(A)** alpha-linolenic acid metabolism, arachidonic acid metabolism, biosynthesis of unsaturated fatty acids; **(B)** glycerophospholipid metabolism; **(C)** galactose metabolism; **(D)** primary bile acid biosynthesis; **(E)** propanoate metabolism, synthesis and degradation of ketone bodies, and butyrate metabolism; **(F)** phenylalanine metabolism; **(G)** tryptophan metabolism, tyrosine metabolism.

Spearman correlation analysis was performed between the metabolites involved in the common metabolic pathways and gut microbiota. Based on two different factors, gut microbiota and metabolites, factors with a correlation coefficient >0.9 were chosen as preliminary targets to investigate the effect of CSPs. In correlation analysis, endogenous metabolites derived from serum are represented as “S-”, and those derived from urine are represented as “U-”. As shown in **Figure 11**, at the phylum level, Firmicutes and Bacteroidota had large correlation coefficients. Increased expression of Firmicutes in DN was negatively correlated with S-a-linolenic acid, S-EETs, S-PC, S-phenylalanine, S-tyrosine, S-tryptophan, U-P-cresol sulfate and U-indoxyl sulfate but positively correlated with U-thromboxane B2, S-LysoPC, S-bile acid, U-acetoacetic acid, U-3-hydroxybutyric acid, S-P-cresol sulfate and S-indoxyl sulfate. Bacteroidota showed an opposite trend to Firmicutes. At the genus level, Lachnospiraceae_NK4A136_group and Dubosiella exhibited low expression levels in the DN and showed the strongest correlation with metabolites. As shown in **Figure 11**, these two genus showed a positive correlation with S-PC, U-taurine, S-taurocholic acid, S-tyrosine, S-tryptophan, S-phenylalanine, U-P-cresol sulfate and U-indoxyl sulfate and a negative correlation with S-LysoPC, S-bile acids, S-3-hydroxybutyric acid, U-oxoglutaric acid, U-acetic acid, galactose metabolism, indole sulfate and p-phenol sulfate in the serum.

## 4 Discussion

The production of chronic low-grade inflammation and the body’s oxidative stress state are key mechanisms underlying the development and progression of diabetic nephropathy, which can directly lead to fibrosis and tissue damage in the kidneys and ultimately result in a gradual deterioration of renal function. There exists an axis of mutual interaction between the gut microbiome and the host liver, pancreas, and kidneys, and their imbalance can result in the entry of endotoxins and bacterial metabolic products into the circulatory system, thereby stimulating the production of inflammatory reactions. Through the study described in this paper, the increased levels of inflammatory factors IL-6 and TNF-a in the serum of diabetic nephropathy rats were observed, as well as abnormal changes in oxidative stress factors SOD and MDA levels, indicating a clear inflammatory response and oxidative stress state in the organism. Investigating the influence of CSPs on the bacterial structure and endogenous metabolites in this state can help us identify advantageous bacterial species and metabolites that are relevant to therapeutic effects.

The ratio of B/F (Bacteroidota/Firmicutes) has been widely recognized as a marker of the disruption of gut microbiota, which is thought to be closely associated with metabolic syndromes such as obesity, diabetes and comorbidities, and constipation. It has been reported in the literature that the proportion of B/F in the gut microbiota is reduced in diabetic patients at different periods, and some papers also mentioned that this imbalance could cause oxidative stress and inflammatory factors in the blood, triggering low-grade systemic chronic inflammation (15, 16). Activated chronic inflammation may cause organ and circulatory collateral damage (17); Therefore, regulating the B/F can be an effective strategy for suppressing inflammation in diabetic nephropathy (18, 19). At the genus level, the correlation between Lachnospiraceae-NK4A136-group and Dubosiella was strong, and both of them significantly reduced in the DN. The Lachnospiraceae-NK4A136 group is currently considered to be a beneficial bacterium, is significantly reduced in patients with different types of kidney injury (20) and is thought to act by three mechanisms. First, it can delay insulin resistance or increase insulin secretion, and intervene before kidney injury occurs (21). Second, it can increase the content of short-chain fatty acids and maintain the integrity of the intestinal barrier (22). Meanwhile, it can inhibit the expression and activity of HDAC (Histone Deacetylase) and reduce the kidney damage caused by the abnormal increase in uremic toxins due to the change in gut microbiota structure (23). In another genus, Dubosiella, some studies suggest that it is a beneficial bacterium that actually produces short-chain fatty acids. Its low expression is closely related to the development of some diseases, such as diabetes (24) and hyperlipidemia (25), and it is expected to become a probiotic widely used in the medical field (26).

**Figure 12:**
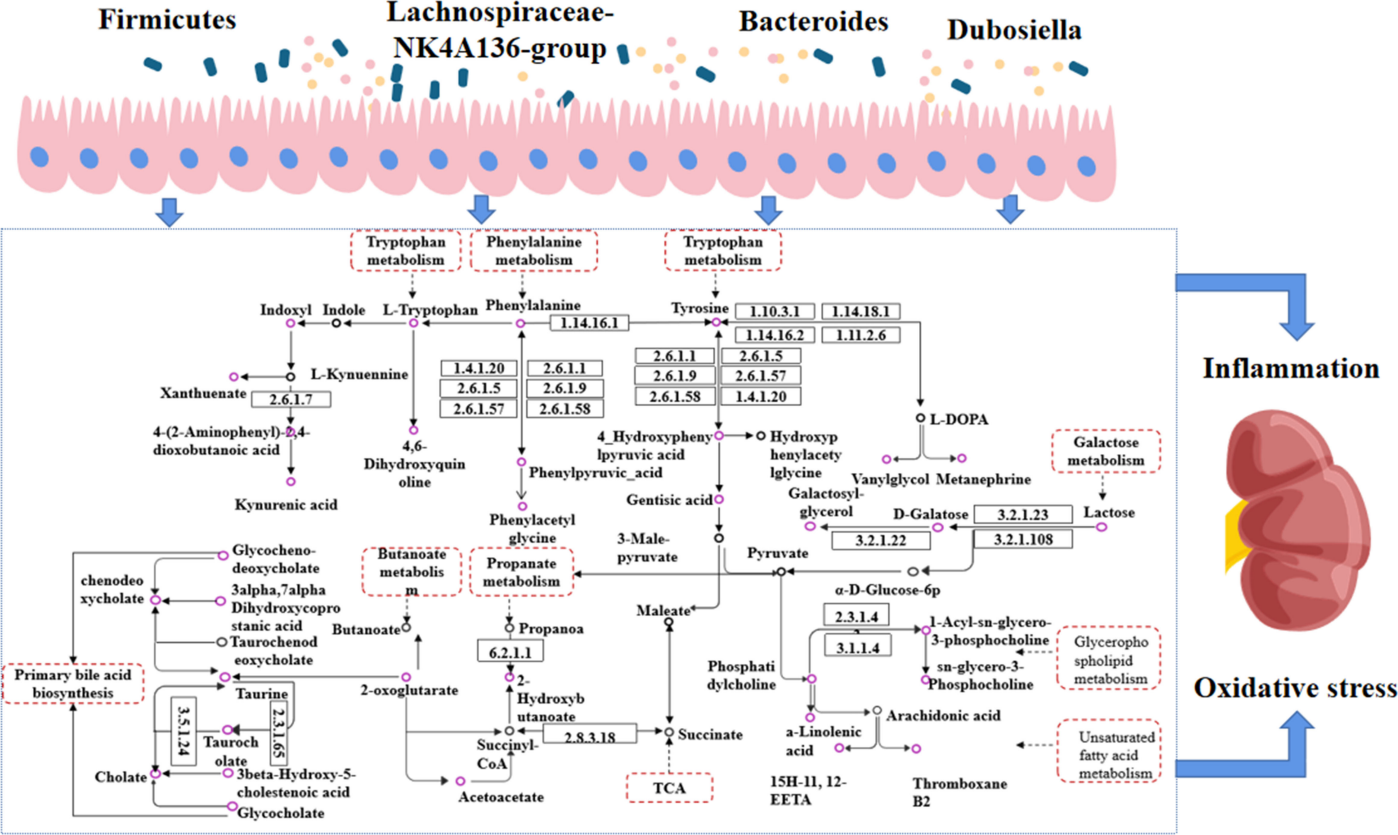
Interaction diagram between gut microbiota and endogenous metabolism. Purple circles represent metabolites with altered levels in CSPs, black circles represent metabolites with unchanged levels; dashed rectangular boxes indicate metabolic pathways.

**Figure 13:**
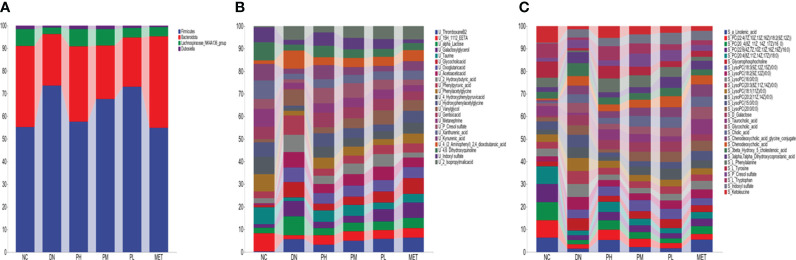
The changes in the content of correlated gut microbiota and endogenous metabolites were observed in each group. **(A)** gut microbiota; **(B)** urine; **(C)** serum.

The endogenous metabolites were closely related to the above phyla and genera based on correlation analysis. The interactive relationships among endogenous metabolites are shown in **Figure 12**, and the relative content changes within each group are depicted in **Figure 13**. U-Thromboxane B2 and U-15H-11,12-EETA (EETs) downstream of arachidonic acid and linolenic acid in unsaturated fatty acids show a strong correlation with the gut microbiota. Current studies suggest that a-linolenic acid (ALA) could stabilize cellular function, resulting in more stable insulin receptor function on the cell membrane (27, 28). Meanwhile, studies have found that ALA can significantly increase the activity of SIRT1 and restore insulin resistance caused by high-level glucose induction (29, 30). At present, it is believed that it has the potential for anti-inflammatory activity, which can prevent high glucose-induced vascular damage (31). Thromboxane B2, one of the metabolites of thromboxane A2, could be used as an indicator of the activity of the thromboxane A2/thromboxane B2 system, the activation of which was associated with an inflammatory response (32, 33). Published studies have confirmed that the level of thromboxane B2 is increased significantly in diabetic nephropathy and is believed to be related to inflammation and oxidative stress (34, 35). EETs, a type of epoxotrienoic acid, are bioactive substances produced by the metabolism of arachidonic acid that have vasodilatory, anti-inflammatory and anti-platelet effects and play an important role in the pathogenesis of diabetic nephropathy (**Figure 12**). EETs can play a role in preventing renal failure by improving renal hemodynamics, inhibiting tubular cell apoptosis, and reducing oxidative stress(36, 37). In the results, it was found that CSPs can modulate multiple inflammation-related biomarkers in diabetic nephropathy rats, including elevated levels of ALA and EETs, and decreased levels of Thromboxane B2, indicating the significant anti-inflammatory effect of CSPs (**Figure 13**).

Another metabolic pathway of strong relevance is galactose metabolism, involving metabolites such as S-galactose, U-galactosylglycerol, and U-alpha-lactose. Galactose can cause oxidative stress in the body and is currently used in the establishment of animal models of aging and oxidative stress. Galactosylglycerol is an intermediate of galactose and glycerolipid metabolism, and its trend changes in line with that of galactose (**Figure 12**). Alpha-lactose is a sugar that is broken down into glucose and galactose during digestion and absorbed through the intestine into the blood circulation to provide the body with an energy supply. Abnormal changes in the gut microbiota or problems with the liver and kidneys may affect the metabolism and excretion of carbohydrates, leading to a large amount of alpha-lactose appearing in the urine. This experimental study found that galactose and galactosylglycerol both increased (**Figures 12**, **13**), and combined with oxidative stress factors, indicating oxidative stress conversion in the model rats, leading to kidney tissue damage. CSPs can modulate galactose metabolism and reduce oxidative stress in the body (**Figure 13**).

S-2-hydroxybutyric-acid and U-acetoacetic acid are highly expressed in urine or blood and are ketone bodies. These substances, stimulated by relatively insufficient insulin secretion or hyperglycemia, were prone to fatty acid oxidation, and in cases of liver and kidney dysfunction, they are more prone to ketoacidosis (38). Another elevated substance is Oxoglutaric acid, which is distributed in the liver and gastrointestinal tract and is involved in various metabolic pathways, including TCA and amino acid synthesis. Dysregulation of this level is closely related to DN (39, 40). The results showed an increased level of Oxoglutaricoxoglutaric acid in urine (**Figures 12**, **13**), suggesting that it may be caused by its decreasedecreased reabsorption by injured renal tissue (41).

Glycerophospholipid metabolites are important mediators of inflammation and oxidative stress and are involved in insulin resistance and hyperglycemia-induced vascular complications such as diabetic nephropathy and diabetic eye disease (42, 43). Phosphatidylcholine (PC) is the main component that affects the stability of cell membranes and is the material basis for cells to exert their physiological functions. LysoPC is an important substance in the glycerophospholipid metabolism pathway (**Figure 12**), and its metabolites are significantly associated with an increased risk of vascular diseases (44). Research found that the ratio of glycerophospholipid/PC is closely related to obesity and the complications induced by high glucose and high fat (45). Similarly, the study conducted by Liu, J et al. (46) found that a lower level of PC is associated with diabetic nephropathy proteinuria levels. The results of this experimental study showed that after treatment with CSPs, the PC level was significantly increased, while glycerophospholipids and different structures of LysoPC were decreased (**Figures 12**, **13**), indicating that CSPs can adjust the microbial structure, affect glycerophospholipid metabolism in the body, and reduce organ cell damage (**Figure 13**).

Bile acid metabolism is most susceptible to the influence of the gut microbiota, and the accumulation of a large amount of bile acid can eventually lead to mitochondrial dysfunction, endoplasmic reticulum stress, tissue cell inflammation, damage and death (47, 48). In particular, Lu, J. et al. (49) showed that conjugated bile acid levels were positively associated with an increased risk of diabetes. It is worth noting that the level of taurine decreased in bile metabolism, a substance currently known to be beneficial in diabetic nephropathy, which has significant antioxidant properties, improves diabetic insulin resistance, reduces the oxidative stress state produced by stimulation of chronically high glucose levels, and reduces comorbidities (50, 51). In this experimental study, it was found that the levels of chenodeoxycholic acid, chenodeoxycholic acid glycine conjugate, cholic acid and glycocholic acid increased, while taurine and Taurocholic acid decreased (**Figures 12**, **13**), suggesting that the structure of the gut microbiota in diabetic nephropathy rats affects the circulation of bile acids in the intestine, leading to stress responses and inflammation in rats.

Chronic kidney impairment is closely related to the metabolism of aromatic amino acids, such as phenylalanine, tyrosine, and tryptophan, including their degradation, synthesis, and excretion. Tyrosine can only be synthesized by hydroxylation of phenylalanine. Studies have shown that the presence of lower serum tyrosine levels in subjects with chronic renal function may be due to the insufficiency of phenylalanine and dysfunction of phenylalanine hydroxylase, resulting in impaired conversion of phenylalanine to tyrosine (52, 53), while the oxidative stress state accompanying the onset of diabetes limits the activity of tetrahydrobiopterin, further damaging phenylalanine hydroxylase activity (54). Meanwhile, there is already evidence to suggest that the level of tryptophan is closely related to various types of chronic kidney injury, showing a negative correlation trend (55–57). It is worth considering that the structure of the gut microbiota changes, and tryptophan, tyrosine, and phenylalanine are metabolized by the microbiota into indole and phenol (**Figure 12**). After sulfation in the liver, they generated uremic toxins such as indoxyl sulfate and p-cresyl sulfate (58, 59). They enter the blood circulation and are excreted by the kidneys, and when the metabolites exceed the metabolic capacity of the body or in patients with kidney injury, they accumulate significantly in the blood and kidneys and cannot be excreted normally through the urine, inducing the production of free radicals in renal tubular cells and thylakoid cells, triggering oxidative stress, enhancing cytokine expression and inflammatory responses, and thus causing cellular damage (58). In this experiment, the levels of phenylalanine, tryptophan, and tyrosine were reduced, while the intestinal bacterial metabolites IS and PCS decreased in urine but increased in serum. These changes in the gut microbiota led us to believe that the metabolism of uremic toxins was accelerated, resulting in kidney tissue damage. Traditional Chinese medicine believes that corn silk has a significant diuretic effect, which is reflected in the experiment results by the urinary excretion of uremic toxins and the storage in serum (**Figure 13**).

## 5 Conclusion

The development of diabetes and diabetic nephropathy, a metabolic disease with many causative factors, is often treated symptomatically with single-target research, which cannot fundamentally delay the progression of the disease. With the continuous updating of detection techniques and theories, a breakthrough was eventually achieved, and researchers discovered that the interaction between gut microbiota and endogenous metabolites compensated for the shortcomings of their respective studies, providing a new understanding for the study of this chronic metabolic disease. In this study, based on the establishment of diabetic nephropathy model rats, 16S RNA amplification and metabolomics were performed using KEGG function prediction to find the overlapping metabolic pathways. Afterward, Spearman correlation analysis was used to find the key dominant strains and the metabolic pathways where the dominant strains function. It was found that Firmicutes, Bacteroidota, Lachnospiraceae-NK4A136-group and Dubosiella in the DN correlated and varied widely, involving glycerophospholipid metabolism, bile acid metabolism, and aromatic amino acid metabolism, possibly due to abnormalities in these metabolic pathways, resulting in LysoPC, bile acids, and uremic toxins IS, and PCS accumulation, the high circulation of these substances *in vivo*, increasing the expression of oxidative stress and chronic low-grade inflammation in the body, causing kidney damage, suggesting that this may be the most critical for diabetic nephropathy. This may be the key mechanism proposed by some researchers to solve diabetic nephropathy by finding the root cause of inflammation and oxidative stress.

As a food and medicine, previous studies have demonstrated that corn silk can improve insulin resistance and lower high blood glucose levels in type 2 diabetes model rats. In the present experimental study, early administration of CSPs intervention significantly modulated the structure of the gut microbiota and endogenous metabolites in rats with diabetic nephropathy and reduced the malignant metabolites circulating within the organism. Perhaps it can be used as an adjunctive therapy for preventing diabetes or preventing the progression of diabetes to diabetic nephropathy. The key is that it should be safe and have no side effects. Of course, this study can only serve as a preliminary exploration of the mechanism of action, and later, fecal microbiota transplantation or intervention with a single strain of bacteria should be carried out to target changes in endogenous metabolites to provide a scientific basis for real-world evaluation, which is what we intend to do.

## Data availability statement

The data presented in the study are deposited in the Figshare, accession number: https://doi.org/10.6084/m9.figshare.24611829.v1.

## Ethics statement

The animal study was approved by The animal ethics committee of the Academy of Traditional Chinese Medicine of Heilongjiang Province ([2011]93). The study was conducted in accordance with the local legislation and institutional requirements.

## Author contributions

WD conceived and performed the study, conducted statistical analyses, generated the figures, and wrote the first draft of the manuscript. YZ, XL and JH participated in the completion of some experiments and data processing. WW conceived of and designed the experiments. All authors contributed to the article and approved the submitted version.
