# Supplementary material for: Corn silk polysaccharides attenuate diabetic nephropathy through restoration of the gut microbial ecosystem and metabolic homeostasis
Source: Front Endocrinol (Lausanne). 2023 Dec 4;14:1232132. doi: 10.3389/fendo.2023.1232132 (PMC10726137; doi:10.3389/fendo.2023.1232132)
Supplement: Supplementary file 1 [file Table_1.docx]

**Supplementary Table 1 Identification results and trends of potential biomarkers in DN rat urine**

| No. | Compound | RT | m/z | Adducts | Formula | Mass Error (ppm) | Fold Change | Highest Mean | Lowest Mean | Vip |
| --- | --- | --- | --- | --- | --- | --- | --- | --- | --- | --- |
| 1 | D-Galactose | 0.93 | 179.0556 | M-H | C6H12O6 | -2.767847738 | 5.832031633 | DN | NC | 4.82133 |
| 2 | Taurine | 0.95 | 124.0072 | M-H | C2H7NO3S | -1.794950495 | 8.42616492 | NC | DN | 2.6507 |
| 3 | alpha-Lactose | 1.02 | 365.1047 | M+Na | C12H22O11 | -2.028332469 | 43.86892665 | DN | NC | 8.88862 |
| 4 | N-Acetyllactosamine | 1.04 | 406.1317 | M+Na | C14H25NO11 | -0.784717276 | 4.945516241 | DN | NC | 1.45225 |
| 5 | Galactosylglycerol | 1.08 | 277.0892 | M+Na | C9H18O8 | -0.584520002 | 4.635450443 | DN | NC | 2.37046 |
| 6 | Oxoglutaric acid | 1.11 | 145.0137 | M-H | C5H6O5 | -3.803381253 | 5.320264619 | DN | NC | 4.60757 |
| 7 | Acetoacetic acid | 1.13 | 101.025 | M-H | C4H6O3 | 2.16672623 | 6.158878602 | DN | NC | 4.04222 |
| 8 | 4-Guanidinobutanoic acid | 1.43 | 146.0915 | M+H | C5H11N3O2 | -3.451920285 | 2.41845691 | NC | DN | 2.3919 |
| 9 | Pipecolic acid | 1.7 | 130.0854 | M+H | C6H11NO2 | -1.476883585 | 2.250188522 | NC | DN | 1.72327 |
| 10 | N-Acetylneuraminic acid 9-phosphate | 1.79 | 390.0794 | M+H | C11H20NO12P | -0.408297434 | 3.208734907 | NC | DN | 1.01726 |
| 11 | Citric acid | 1.83 | 191.0195 | M-H | C6H8O7 | -1.227011154 | 2.681344507 | DN | NC | 10.6248 |
| 12 | Deoxyadenosine monophosphate | 2.49 | 332.0782 | M+H | C10H14N5O6P | 3.192121653 | 18.18986363 | DN | NC | 1.04429 |
| 13 | 3-Hydroxymethylglutaric acid | 2.68 | 161.045 | M-H | C6H10O5 | -3.473434877 | 2.985626457 | DN | NC | 1.98877 |
| 14 | 3,4-Dihydroxyphenylglycol O-sulfate | 4.82 | 249.0069 | M-H | C8H10O7S | -2.247768882 | 2.965143484 | NC | DN | 3.13585 |
| 15 | Adipic acid | 4.84 | 145.0502 | M-H | C6H10O4 | -3.256598601 | 8.992967446 | DN | NC | 1.70672 |
| 16 | Thromboxane B2 | 5.28 | 393.2229 | M+Na | C20H34O6 | -0.933290452 | 2.268430361 | DN | NC | 1.87759 |
| 17 | Hydroxyphenylacetylglycine | 5.39 | 208.0608 | M-H | C10H11NO4 | -3.389313038 | 2.166942001 | NC | DN | 1.45482 |
| 18 | Xanthurenic acid | 5.47 | 204.0299 | M-H | C10H7NO4 | -1.819686957 | 3.760771799 | NC | DN | 3.69732 |
| 19 | 2-Isopropylmalicacid | 5.78 | 175.0609 | M-H | C7H12O5 | -1.934185965 | 5.085925849 | DN | NC | 1.96709 |
| 20 | Kynurenic acid | 5.96 | 190.0492 | M+H | C10H7NO3 | -3.545764503 | 7.143337662 | DN | NC | 2.39153 |
| 21 | Hydroxyphenylpyruvic acid | 6.05 | 179.0348 | M-H | C9H8O4 | -1.208549881 | 5.078100636 | NC | DN | 1.65414 |
| 22 | Vanylglycol | 6.35 | 185.0801 | M+H | C9H12O4 | -4.025043924 | 3.773811689 | DN | NC | 1.02115 |
| 23 | 4-(2-Aminophenyl)-2,4-dioxobutanoic acid | 6.37 | 206.0456 | M-H | C10H9NO4 | -1.303251754 | 90.29249536 | DN | NC | 3.32884 |
| 24 | Hippuric acid | 6.57 | 178.0509 | M-H | C9H9NO3 | -0.322686521 | 2.135281917 | NC | DN | 5.07816 |
| 25 | Pimelic acid | 6.61 | 159.066 | M-H | C7H12O4 | -1.978577751 | 2.157140654 | DN | NC | 1.63571 |
| 26 | 3-Hydroxydodecanedioic acid | 6.73 | 245.1392 | M-H | C12H22O5 | -0.887689082 | 4.477395085 | NC | DN | 2.70477 |
| 27 | Riboflavin | 7.11 | 376.1383 | M+H | C17H20N4O6 | 0.145345485 | 7.130669452 | NC | DN | 7.76384 |
| 28 | Phenylpyruvic acid | 7.13 | 163.0394 | M-H | C9H8O3 | -1.157407032 | 3.124756152 | DN | NC | 2.23934 |
| 29 | a-Linolenic acid | 7.15 | 301.2122 | M+Na | C18H30O2 | -1.813105481 | 5.133184567 | NC | DN | 2.69953 |
| 30 | Indoxyl sulfate | 7.27 | 212.0023 | M-H | C8H7NO4S | -0.065444199 | 2.031060156 | NC | DN | 14.4993 |
| 31 | 4,6-Dihydroxyquinoline | 7.39 | 162.054 | M+H | C9H7NO2 | -5.926214319 | 10.92065236 | NC | DN | 5.69415 |
| 32 | Phenylacetylglycine | 7.41 | 192.0661 | M-H | C10H11NO3 | -2.755778365 | 2.346452234 | NC | DN | 3.29097 |
| 33 | Gentisic acid | 8.45 | 153.0183 | M-H | C7H6O4 | -3.701050316 | 2.27944504 | NC | DN | 3.26075 |
| 34 | P-Cresol sulfate | 8.82 | 187.0069 | M-H | C7H8O4S | -0.353774516 | 3.282609919 | NC | DN | 2.8906 |
| 35 | Tiglylcarnitine | 9.2 | 242.1399 | M-H | C12H21NO4 | 0.347936235 | 3.424512676 | NC | DN | 1.1126 |
| 36 | Indolelactic acid | 9.66 | 204.0666 | M-H | C11H11NO3 | -0.227507706 | 9.654434743 | NC | DN | 3.05984 |
| 37 | Benzoic acid | 9.68 | 121.0299 | M-H | C7H6O2 | 3.240499296 | 2.0149424 | NC | DN | 1.70164 |
| 38 | Metanephrine | 10.25 | 196.0977 | M-H | C10H15NO3 | -0.98967002 | 16.23604725 | NC | DN | 3.04516 |
| 39 | 3-Methylindole | 10.31 | 130.0661 | M-H | C9H9N | -1.286942142 | 3.149678713 | NC | DN | 1.6439 |
| 40 | Sebacic acid | 11.01 | 201.1138 | M-H | C10H18O4 | 2.806873615 | 2.306752154 | DN | NC | 2.72189 |
| 41 | Dodecanedioic acid | 11.32 | 229.1443 | M-H | C12H22O4 | -0.921307628 | 6.307722267 | DN | NC | 2.75349 |
| 42 | cis-4-Hydroxycyclohexylacetic acid | 11.4 | 157.0864 | M-H | C8H14O3 | -4.081686247 | 3.307892714 | NC | DN | 2.28779 |
| 43 | Aldosterone | 11.56 | 361.2013 | M-H | C21H30O5 | -2.040543083 | 21.39537565 | DN | NC | 1.11507 |
| 44 | Cortisone | 11.62 | 361.2008 | M+H | C21H28O5 | -0.371460004 | 3.118601642 | DN | NC | 1.24126 |
| 45 | Glycocholic acid | 11.83 | 464.3017 | M-H | C26H43NO6 | -0.060942547 | 17.26191978 | DN | NC | 1.26982 |
| 46 | 7-Sulfocholic acid | 11.93 | 487.2375 | M-H | C24H40O8S | 0.749022917 | 3.792435027 | DN | NC | 4.22011 |
| 47 | 15H-11,12-EETA | 14.06 | 337.2372 | M+H | C20H32O4 | -0.502713737 | 8.239659557 | NC | DN | 2.04094 |
