# Supplementary material for: Corn silk polysaccharides attenuate diabetic nephropathy through restoration of the gut microbial ecosystem and metabolic homeostasis
Source: Front Endocrinol (Lausanne). 2023 Dec 4;14:1232132. doi: 10.3389/fendo.2023.1232132 (PMC10726137; doi:10.3389/fendo.2023.1232132)
Supplement: Supplementary file 2 [file Table_2.docx]

**Supplementary Table 2 Identification results and trends of potential biomarkers in DN rat serum**

| No. | Compound | Rt | m/z | Adducts | Formula | Mass Error (ppm) | Fold Change | Highest Mean | Lowest Mean | VIP |
| --- | --- | --- | --- | --- | --- | --- | --- | --- | --- | --- |
| 1 | Glycerophosphocholine | 0.89 | 280.0915 | M+Na | C8H20NO6P | -1.983320598 | 2.304327868 | DN | NC | 1.38708 |
| 2 | D-Galactose | 0.91 | 203.0517 | M+Na | C6H12O6 | -4.89709295 | 1.945572595 | DN | NC | 2.87095 |
| 3 | Oxidized glutathione | 1.72 | 611.1449 | M-H | C20H32N6O12S2 | 0.420675279 | 2.684480499 | NC | DN | 1.3498 |
| 4 | L-Tyrosine | 1.78 | 180.0662 | M-H | C9H11NO3 | -2.192336564 | 2.652296123 | NC | DN | 1.85501 |
| 5 | 2-Hydroxybutyric acid | 2.12 | 103.041 | M-H | C4H8O3 | 3.686988389 | 2.207080779 | DN | NC | 1.38252 |
| 6 | Hexanoylglycine | 2.29 | 172.0975 | M-H | C8H15NO3 | -2.183147481 | 3.176394955 | DN | NC | 1.31281 |
| 7 | L-Phenylalanine | 2.41 | 164.0717 | M-H | C9H11NO2 | 0.037530985 | 1.301661699 | NC | DN | 1.18095 |
| 8 | L-Tryptophan | 2.69 | 203.0826 | M-H | C11H12N2O2 | -0.156186712 | 1.681661771 | NC | DN | 2.76257 |
| 9 | Taurochenodesoxycholic acid | 2.87 | 500.3058 | M+H | C26H45NO6S | 3.624975763 | 2.101244989 | NC | DN | 1.30208 |
| 10 | Pyrocatechol sulfate | 2.94 | 188.9857 | M-H | C6H6O5S | -3.140334162 | 5.590140617 | DN | NC | 1.44779 |
| 11 | Ketoleucine | 3.14 | 129.0553 | M-H | C6H10O3 | -3.353449169 | 2.244508843 | NC | DN | 1.15017 |
| 12 | Indoxyl sulfate | 3.24 | 212.0017 | M-H | C8H7NO4S | -2.928126169 | 2.454114553 | DN | NC | 6.23044 |
| 13 | 2-Methylhippuric acid | 3.55 | 192.0661 | M-H | C10H11NO3 | -2.757500387 | 5.353148329 | NC | DN | 1.10529 |
| 14 | P-Cresol sulfate | 3.65 | 187.0067 | M-H | C7H8O4S | -1.754602501 | 5.496336858 | DN | NC | 4.18213 |
| 15 | Equol 7-O-glucuronide | 3.74 | 417.1182 | M-H | C21H22O9 | -2.280583315 | 2.23466875 | NC | DN | 1.67997 |
| 16 | PC(18:2(9Z,12Z)/P-18:1(9Z)) | 4.26 | 768.589 | M+H | C44H82NO7P | -1.498853286 | 2.501765693 | NC | DN | 1.49449 |
| 17 | PC(18:2(9Z,12Z)/P-18:1(11Z)) | 4.26 | 768.589 | M+H | C44H82NO7P | -1.498853286 | 2.501765693 | NC | DN | 1.49449 |
| 18 | Taurocholic acid | 4.53 | 514.2834 | M-H | C26H45NO7S | -1.87584974 | 4.017898387 | NC | DN | 4.55619 |
| 19 | Deoxycholic acid 3-glucuronide | 4.61 | 567.3165 | M-H | C30H48O10 | -1.716293443 | 33.84130387 | DN | NC | 6.10202 |
| 20 | Bilirubin | 4.7 | 585.2704 | M+H | C33H36N4O6 | -0.635772512 | 1.353598194 | NC | DN | 1.27103 |
| 21 | Glycocholic acid | 4.77 | 464.301 | M-H | C26H43NO6 | -1.722414715 | 2.741985076 | DN | NC | 6.744 |
| 22 | Cholic acid | 4.87 | 407.279 | M-H | C24H40O5 | -3.089631683 | 2.968914034 | DN | NC | 6.04789 |
| 23 | PC(22:4(7Z,10Z,13Z,16Z)/18:2(9Z,12Z)) | 4.96 | 856.5829 | M+Na | C48H84NO8P | 0.267903726 | 2.922345175 | NC | DN | 1.11499 |
| 24 | PC(18:2(9Z,12Z)/22:4(7Z,10Z,13Z,16Z)) | 4.96 | 856.5829 | M+Na | C48H84NO8P | 0.267903726 | 2.922345175 | NC | DN | 1.11499 |
| 25 | Chenodeoxycholic acid glycine conjugate | 5.05 | 448.3057 | M-H | C26H43NO5 | -2.608598038 | 2.677055443 | DN | NC | 1.56194 |
| 26 | Chenodeoxycholic acid | 5.77 | 391.2846 | M-H | C24H40O4 | -2.10433615 | 3.049917508 | DN | NC | 2.49571 |
| 27 | 9,10-DHOME | 6.08 | 313.2377 | M-H | C18H34O4 | -2.17525924 | 1.777793769 | DN | NC | 2.03941 |
| 28 | Sphingosine 1-phosphate | 6.20 | 378.2407 | M-H | C18H38NO5P | -2.119450904 | 1.165134119 | NC | DN | 2.18212 |
| 29 | LysoPC(14:0/0:0) | 6.26 | 467.3006n | M+H | C22H46NO7P | -1.256777226 | 1.675104798 | DN | NC | 1.99708 |
| 30 | Docosatrienoic acid | 6.30 | 357.2784 | M+Na | C22H38O2 | 3.022962119 | 5.862207538 | DN | NC | 3.07088 |
| 31 | LysoPC(18:3(9Z,12Z,15Z)/0:0) | 6.39 | 518.3235 | M+H | C26H48NO7P | -1.267323721 | 1.8708764 | DN | NC | 2.67613 |
| 32 | 12(13)Ep-9-KODE | 6.5 | 309.2063 | M-H | C18H30O4 | -2.767099841 | 2.471737106 | DN | NC | 2.67695 |
| 33 | LysoPC(18:2(9Z,12Z)/0:0) | 6.91 | 542.3209 | M+Na | C26H50NO7P | -1.520279995 | 1.465689517 | DN | NC | 2.60427 |
| 34 | Octadecenoylcarnitine | 7.00 | 426.3575 | M+H | C25H47NO4 | -0.729588859 | 2.138599976 | DN | NC | 1.4153 |
| 35 | LysoPC(16:0/0:0) | 7.19 | 495.3321 | M+H | C24H50NO7P | -0.687208518 | 1.232852035 | DN | NC | 5.13573 |
| 36 | LysoPC(20:3(8Z,11Z,14Z)/0:0) | 7.30 | 546.355 | M+H | C28H52NO7P | -0.747491034 | 3.056356792 | DN | NC | 9.38991 |
| 37 | LysoPC(20:3(5Z,8Z,11Z)/0:0) | 7.30 | 546.355 | M+H | C28H52NO7P | -0.747491034 | 3.056356792 | DN | NC | 9.38991 |
| 38 | LysoPC(18:1(11Z)/0:0) | 7.80 | 522.3551 | M+H | C26H52NO7P | -0.579490255 | 1.657894216 | DN | NC | 15.7291 |
| 39 | LysoPC(18:1(9Z)/0:0) | 7.80 | 522.3551 | M+H | C26H52NO7P | -0.579490255 | 1.657894216 | DN | NC | 15.7291 |
| 40 | Stearoylcarnitine | 7.95 | 428.3734 | M+H | C25H49NO4 | -0.056138043 | 4.992399222 | DN | NC | 2.2928 |
| 41 | Stearic acid | 7.96 | 283.2638 | M-H | C18H36O2 | -1.607532844 | 1.550893105 | DN | NC | 1.17986 |
| 42 | LysoPC(20:2(11Z,14Z)/0:0) | 8.18 | 547.3633 | M+H | C28H54NO7P | -0.829715919 | 1.57363843 | DN | NC | 2.99776 |
| 43 | 9,10-Epoxyoctadecenoic acid | 8.45 | 295.2271 | M-H | C18H32O3 | -2.60876133 | 1.838330141 | DN | NC | 1.23857 |
| 44 | LysoPC(15:0/0:0) | 9.35 | 480.3084 | M-H | C23H48NO7P | -2.391585164 | 1.975285459 | DN | NC | 5.58215 |
| 45 | LysoPC(18:0/0:0) | 9.37 | 523.3633n | M+H | C26H54NO7P | -0.888208414 | 1.227520322 | DN | NC | 12.1035 |
| 46 | Retinyl ester | 9.95 | 301.2167 | M-H | C20H30O2 | -2.061045437 | 1.566352391 | NC | DN | 1.06268 |
| 47 | alpha-Linolenic acid | 10.44 | 277.2168 | M-H | C18H30O2 | -1.975301332 | 2.180208836 | NC | DN | 1.19838 |
| 48 | Docosahexaenoic acid | 10.87 | 327.2325 | M-H | C22H32O2 | -1.373486924 | 1.53656386 | NC | DN | 3.48608 |
| 49 | Arachidonic acid | 11.37 | 303.2327 | M-H | C20H32O2 | -0.933247583 | 1.33932656 | NC | DN | 3.44792 |
| 50 | PC(20:4(8Z,11Z,14Z,17Z)/16:0) | 11.77 | 804.5516 | M+Na | C44H80NO8P | 0.333981749 | 2.589536052 | NC | DN | 5.794 |
| 51 | PC(20:4(5Z,8Z,11Z,14Z)/16:0) | 11.77 | 804.5516 | M+Na | C44H80NO8P | 0.333981749 | 2.589536052 | NC | DN | 5.794 |
| 52 | LysoPC(20:0/0:0) | 11.98 | 551.3945 | M+H | C28H58NO7P | -1.063209278 | 1.462358326 | DN | NC | 1.55573 |
| 53 | Docosapentaenoic acid (22n-3) | 12.35 | 329.2479 | M-H | C22H34O2 | -2.076168488 | 1.91662329 | NC | DN | 1.12541 |
| 54 | Adrenic acid | 12.98 | 331.2636 | M-H | C22H36O2 | -1.869709087 | 2.672409961 | NC | DN | 1.08035 |
| 55 | PC(22:6(4Z,7Z,10Z,13Z,16Z,19Z)/16:0) | 13.13 | 806.5689 | M+H | C46H80NO8P | -0.602095913 | 3.019788439 | NC | DN | 20.7969 |
| 56 | PC(20:4(8Z,11Z,14Z,17Z)/18:0) | 14.52 | 810.6006 | M+H | C46H84NO8P | -0.219593774 | 2.396990018 | NC | DN | 9.64128 |
| 57 | PC(20:4(5Z,8Z,11Z,14Z)/18:0) | 14.52 | 810.6006 | M+H | C46H84NO8P | -0.219593774 | 2.396990018 | NC | DN | 9.64128 |
